# A New Biomimetic Composite Structure with Tunable Stiffness and Superior Toughness via Designed Structure Breakage

**DOI:** 10.3390/ma13030636

**Published:** 2020-01-31

**Authors:** Xiaohan Wang, Dongxu Li

**Affiliations:** College of Aerospace Science and Engineering, National University of Defense Technology, Changsha 410073, China; lidongxu@nudt.edu.cn

**Keywords:** biomimetic composite structure, sacrificial bond mimicking, tunable stiffness, superior structure toughness

## Abstract

Mimicking natural structures has been highly pursued recently in composite structure design to break the bottlenecks in the mechanical properties of the traditional structures. Bone has a remarkable comprehensive performance of strength, stiffness and toughness, due to the intricate hierarchical microstructures and the sacrificial bonds within the organic components. Inspired by the strengthening and toughening mechanisms of cortical bone, a new biomimetic composite structure, with a designed progressive breakable internal construction mimicking the sacrificial bond, is proposed in this paper. Combining the bio-composite staggered plate structure with the sacrificial bond-mimicking construction, our new structure can realize tunable stiffness and superior toughness. We established the constitutive model of the representative unit cell of our new structure, and investigated its mechanical properties through theoretical analysis, as well as finite element modeling (FEM) and simulation. Two theoretical relations, respectively describing the elastic modulus decline ratio and the unit cell toughness promotion, are derived as functions of the geometrical parameters and the material parameters, and validated by simulation. We hope that this work can lay the foundation for the stiffness tunable and high toughness biomimetic composite structure design, and provide new ideas for the development of sacrificial bond-mimicking strategies in bio-inspired composite structures.

## 1. Introduction

Biological materials, such as bone in mammals and nacre in shells, have attracted constant research interest for their excellent combination of strength, stiffness and toughness [1,2,3,4,5]. For example, bone is a composite material made up of collagen (30%–45% by volume), apatite crystals (30%–50% by volume) and small amounts of non-collagenous proteins [6,7]. The Young’s modulus is about 10 GPa, and the tensile strength is about 80–120 MPa [6,7,8,9]. The key reason for its excellent mechanical performance owes to the “staggered lamellae layer”, an intricate hierarchical micro-structure inside the cortical bone [10,11,12,13,14]. Besides this, it has also been revealed that the sacrificial bonds within the organic components are among the important factors which account for its excellent energy dissipating mechanisms [15,16,17,18,19]. Sacrificial bonds are defined as relatively weak bonds (and often reversible) that rupture before strong bonds fail under deformation [20]. A load applied to the biological materials would lead to the rupture of sacrificial bonds, which means a huge amount of energy dissipation and a promotion of the toughness of the biological. Meanwhile, due to the retention of strong bonds, the strength of the biological materials would rarely be influenced.

Jäger et al. [7] first proposed a two-dimensional model to mimic the “staggered lamellae layer” microstructure within bone, in which hard plates were staggered in soft matrix. This simplified “biomimetic staggered structure” was also proved to be effective, mimicking other biological materials, such as nacre and spider silk fibers, and then improved and widely used in the design and analysis of biomimetic structures. Most optimizations of the classic “biomimetic staggered structure” focus on the shape and size of the constituent part. Gao et al. [21,22] firstly established a simplified one-dimensional analytic model to analyze the mechanical properties of the “biomimetic staggered structure”. The critical “overlapped length” was derived, which was proven as a crucial structure parameter affecting biological structural strength and toughness. Kim et al. [23] proposed an extended “shear-lag” analytic model for the elastic properties of the “biomimetic staggered structure”. With their more detailed analysis of the stress distribution in the “biomimetic staggered structure”, they reached the conclusion that the mechanical weak points of this structure should be located at the “middle point of the hard plate” and the “joint of the two hard plates”. Inspired by the diamond-shaped micro-pores in the cortical bone, Hao et al. [24,25] proposed a new “biomimetic staggered structure” with optimized geometric configuration of the “hard plate”, and established its constitutive model. Through theoretical analysis and FEM simulation, they showed that this new biomimetic structure could eliminate the stress concentration at the middle point of the hard plate. Wei et al. [26] considered how the ductility of the matrix in shear deformation plays an important role in the mechanical properties of the “biomimetic staggered structure”. They first presented an analytical model taking the matrix plasticity and failure into account. With rigorous derivation, their analytical model predicts characteristic overlap lengths that optimize the mechanical performance in a variety of natural materials with very different geometric structures and at a range of different length scales. Their analytical model found a good agreement compared with experimental measurements of three natural materials, i.e., nacre, collagen molecules, and spider silk fibers.

However, as we mentioned before, the simultaneous high toughness and strength of the cortical bone not only related to its microstructure configuration, but also to its unique biological function, such as the “sacrifice bond”. In our study about the toughening mechanism of the cortical bone, [14] the influence of micro-structure and micro-crack within the “staggered lamellae layer” was especially analyzed. At a micrometer scale it was found that some tiny cracks, appearing under load, can help dissipating energy and lead to the toughness promotion in the direction perpendicular to the micro-crack [14,27,28,29]. This meso-scale phenomenon is similar to the “sacrificial bonds properties” at the molecular scale, to some extent. Therefore, adding a biological function to the classic “biomimetic staggered structure” is a promising design and optimization strategy.

**Figure 1:**
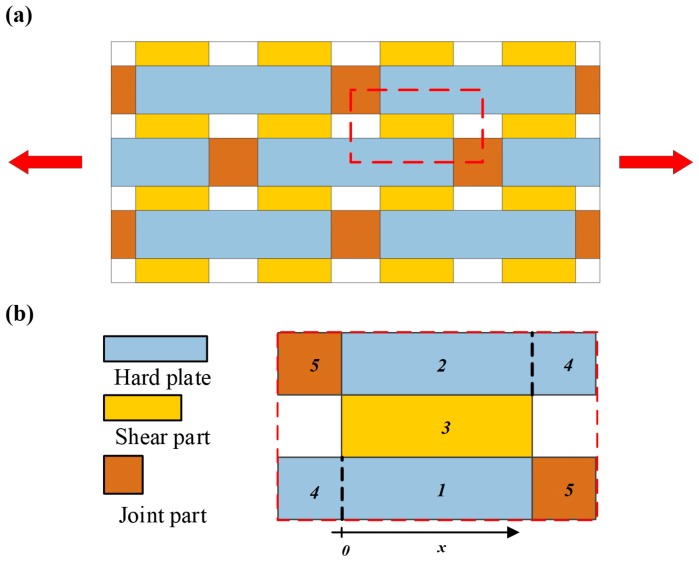
(**a**) Schematic image of our biomimetic composite structure under uniaxial tensile load. (**b**) The unit cell defined in this paper, with the region partition 1 to 5 and coordinate definitions.

Inspired by the hierarchical micro-structure of cortical bone and the “sacrificial bonds” phenomena, we took advantage of the breakage of the mechanical weak points, i.e., the “joint of the two hard plates” to realize biological function. As shown in Figure 1, we proposed a new biomimetic composite structure mimicking the “sacrificial bonds” phenomena, and established its mechanical model. This new “biomimetic staggered structure” could dissipate part of the work of the external load with the designed progressive breakage of the “joint part”, while maintaining the bearing capacity to some extent. Therefore, this new “biomimetic staggered structure” can realize superior structure toughness and the tunable structure stiffness.

With the established constitutive model of the unit cell of our new “biomimetic staggered structure”, we studied the relationship between its mechanical properties and its structural parameters. Although we have not built a laboratory sample of the new “biomimetic staggered structure” for experimental testing, an FEM simulation was carried out to verify the effectiveness of the designed structure. The FEM simulation results agreed well with our theoretical analysis. Ultimately, we wish to put forward a new design and optimization idea for meso/macro-scale biological structures, mimicking “sacrificial bonds” to achieve superior toughness and tunable stiffness mechanical properties.

## 2. Mechanical Model and Theoretical Analysis

Considering the periodicity and symmetry of our structure, we take a unit cell, as defined in Figure 1b, to establish the constitutive model, and further analyze the trends in structure parameters and mechanical response. We defined two phases for a unit cell, “phase 0”: before the “joint part” breakage; and “phase 1” after the “joint part” breakage. With the designed progressive breakage happening under load, the unit cells would change from “phase 0” to “phase 1” one by one. These will reflect the structure parameters (such as stiffness and toughness) of the whole biomimetic structure changing gradually. 

### 2.1. Mechanical Model of Unit Cell

#### 2.1.1. Unit Cell at “Phase 0”

**Figure 2:**
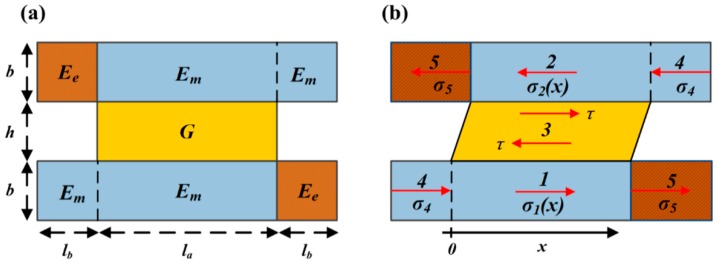
Schematic image of the unit cell at “phase 0”. (**a**) The geometry and material parameters are defined on the undeformed unit cell. (**b**) The region partition 1 to 5 is defined on the deformed unit cell, where Regions 1, 2 and 4 are “hard plate” material, region 3 is “share part” material and Region 5 is “joint part” material. $\sigma_{i}\;\left(i=1,\;2,\;4,\;5\right)$ and τ are respectively, the normal and shear stress distributed in every region, under uniaxial tensile stress. Origin “o” and “x-axis” is the coordinate defined while establishing the constitutive model.

The geometry and material parameters of a unit cell at “phase 0” are shown in Figure 2a. Stress definitions and distribution in a deformed unit cell under uniaxial tensile load, with region partition, are shown in Figure 2b. In a unit cell at “phase 0”, there are “hard plate” (in Regions 1,2 and 4), “shear part” (in Region 3), and “joint part” (in Region 5). We make the simplification that “hard plate” and “joint part” can only hold normal stress, while “shear part” only hold shear stress under uniaxial tensile load. We assume that all parts maintain the characteristics of linear elastic deformation. Moreover, under our design the “joint part” is the only breakable region, and the fracture form is brittle fracture. Therefore, the “maximum tensile stress principle” was taken as the failure criterion for the “joint part”.

As defined in Figure 2a, dimension b is the half thickness of “hard plate” and “joint part”, h is the thickness of the shear part; $l_{a}$ is the length of the shear part (also the uniformly overlapped length of the hard plate); $2l_{a}$ is the length of the “joint part” (also the non-overlapped length of the hard plate). In this study, a uniformly overlapped structure assumption is due to the more efficient load transfer capability [30,31]. The tensile modulus for “hard plate” and “joint part” are denoted as $E_{m}$ and $E_{e}$; while the shear modulus for “region 3” are denoted as G. To easily describine the mechanical model, we also defined several non-dimensional geometrical and material parameters. The geometrical parameters are: the “hard plate” overlapped length to thickness ratio $\rho =l_{a}/b$; the “Region 3” length to thickness ratio $\lambda =l_{a}/h$; the “hard plate” overlapped length to non-overlapped length ratio $\eta =l_{a}/l_{b}$; and the approximate volume fraction of “hard plate” ϕ=2b/(2b+h). The material parameters are: the “joint part” tensile modulus to “hard plate” tensile modulus $\alpha =E_{e}/E_{m}$; and the “Region 3” shear modulus to “hard plate” tensile modulus $\beta =G/E_{m}$.

At “phase 0”, under the coordinate defined in Figure 2b, we established the constitutive model of the unit cell, applying the well-known ‘shear-lag’ model [26].

(1) $$\left\{ \begin{array}{l} b\frac{d\sigma_{1}\left(x\right)}{dx}=-\tau \\ b\frac{d\sigma_{2}\left(x\right)}{dx}=\tau \\ \tau =\frac{G}{h}\left(u_{2}\left(x\right)-u_{1}\left(x\right)\right) \end{array}\right.$$

where the subscripts stand for the region number.

When considering the boundary conditions, because of the uniformly overlapped staggered configuration, we assumed that stress in Region 4 ($\sigma_{4}$) and Region 5 ($\sigma_{5}$) were both uniform. Under this assumption, a boundary condition can be written as

(2) $$\sigma_{1}\left(0\right)=\sigma_{2}\left(l_{a}\right)=\sigma_{4}$$

The other boundary condition was obtained by considering the force equilibrium between Region 5 and the left end of Region 2 (or the right end of Region 1).

(3) $$\sigma_{1}\left(l_{a}\right)=\sigma_{2}\left(0\right)=\sigma_{5}=\frac{E_{e}hb}{Gl_{b}}\frac{d\sigma_{2}\left(0\right)}{dx}+\frac{E_{e}}{E_{m}}\sigma_{4}$$

From the force equilibrium of the whole unit cell, $\sigma_{4}$ and $\sigma_{5}$ should correspond to the following equation

(4) $$\frac{\phi }{2}\left(\sigma_{4}+\sigma_{5}\right)=\bar{\sigma }$$

where ϕ=2b/(2b+h) is the approximate volume fraction of “hard plate”, and $\bar{\sigma }$ is the volume-averaged stress in the unit cell under uniaxial tensile load.

Solving Equation (1) with boundary conditions Equations (2) and (3), and simplified with Equation (4); we obtained the stress distribution in Regions 1,2 and 3, at “phase 0”:

(5) $$\left\{ \begin{array}{l} \sigma_{1}\left(x\right)=\frac{\bar{\sigma }}{\phi }\left(1+\frac{\left(\alpha -1\right)K\sinh\left(K\frac{2x-l_{a}}{l_{a}}\right)}{\alpha \eta \cosh\left(K\right)+\left(\alpha +1\right)K\sinh\left(K\right)}\right)\\ \sigma_{2}\left(x\right)=\frac{\bar{\sigma }}{\phi }\left(1-\frac{\left(\alpha -1\right)K\sinh\left(K\frac{2x-l_{a}}{l_{a}}\right)}{\alpha \eta \cosh\left(K\right)+\left(\alpha +1\right)K\sinh\left(K\right)}\right)\\ \tau \left(x\right)=\frac{\bar{\sigma }}{\phi }\left(\frac{\left(1-\alpha \right)\beta \lambda \cosh\left(K\frac{2x-l_{a}}{l_{a}}\right)}{\alpha \eta \cosh\left(K\right)+\left(\alpha +1\right)K\sinh\left(K\right)}\right) \end{array}\right.$$

where $\alpha =E_{e}/E_{m},\;\beta =G/E_{m},\;\rho =l_{a}/b,\;\lambda =l_{a}/h,\;\eta =l_{a}/l_{b}$ are the non-dimensional geometrical or material parameters defined before; K is a non-dimensional parameter that represents the complex geometrical and material effects of Regions 1, 2 and 3.

(6) $$K=\sqrt{\frac{Gl_{a}{}^{2}}{2E_{m}bh}}=\sqrt{\frac{\beta \rho \lambda }{2}}$$

Combining Equations (2)–(4), we obtained the uniform stress in Regions 4 and 5.

(7) $$\left\{ \begin{array}{l} \sigma_{4}=\frac{\bar{\sigma }}{\phi }\left(\frac{\alpha \eta +2K\tanh\left(K\right)}{\alpha \eta +\left(\alpha +1\right)K\tanh\left(K\right)}\right)\\ \sigma_{5}=\frac{\bar{\sigma }}{\phi }\left(\frac{\alpha \eta +2\alpha K\tanh\left(K\right)}{\alpha \eta +\left(\alpha +1\right)K\tanh\left(K\right)}\right) \end{array}\right.$$

#### 2.1.2. Unit Cell at “Phase 1”

**Figure 3:**
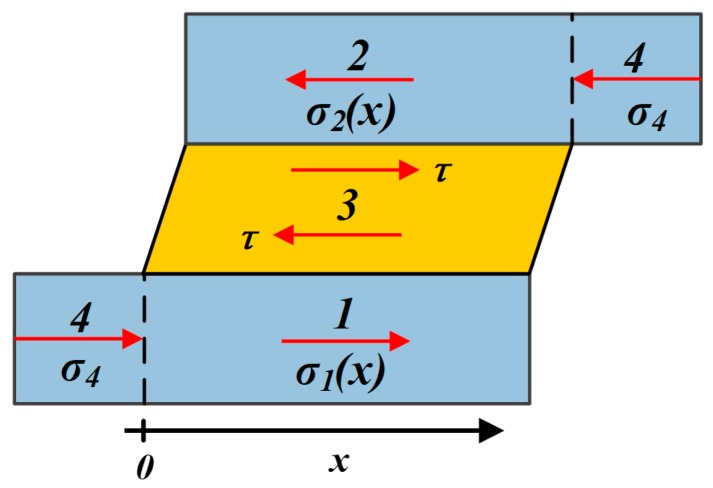
Schematic image of the deformed unit cell at “phase 1”. The geometry and material parameters are inherited from Figure 2. (a) Stress only distributed in Regions 1, 2, 3, 4 because of the breakage of Region 5. Origin “o” and “x-axis” represent the coordinate.

Next, we established the changed constitutive model of a unit cell at “phase 1”, also under the uniaxial tensile load. In the unit cell at “phase 1”, the Region 5 was breaking under deformation and there was only “hard plate” (in Regions 1,2 and 4) and “shear part” (in Region 3), as shown in Figure 3. Moreover, all the geometrical and material parameters of the unit cell can be inherited from “phase 0”.

For unity of the analysis, we used the “shear-lag” model in Regions 1, 2, 3 and then determined the uniform $\sigma_{4}$. Therefore, under the coordinate defined in Figure 3, the shear-lag model in Regions 1, 2 and 3 should still be written as Equation (1). 

However, the boundary conditions would change as the following:

(8) $$\sigma_{1}\left(0\right)=\sigma_{2}\left(l_{a}\right)=\sigma_{4}$$

(9) $$\sigma_{1}\left(l_{a}\right)=\sigma_{2}\left(0\right)=0$$

Moreover, from the force equilibrium of the whole unit cell, $\sigma_{4}$ should be derived as

(10) $$\sigma_{4}=\frac{2}{\phi }\bar{\sigma }$$

Solving Equation (1) with boundary conditions Equations (8) and (9), and simplified with Equation (10), we obtained the stress distribution in Regions 1, 2 and 3 at “phase 1”.

(11) $$\left\{ \begin{array}{l} \sigma_{1}\left(x\right)=\frac{\bar{\sigma }}{\phi }\left(1+\cosh\left(K\frac{2x}{l_{a}}\right)-\coth\left(K\right)\sinh\left(K\frac{2x}{l_{a}}\right)\right)\\ \sigma_{2}\left(x\right)=\frac{\bar{\sigma }}{\phi }\left(1-\cosh\left(K\frac{2x}{l_{a}}\right)+\coth\left(K\right)\sinh\left(K\frac{2x}{l_{a}}\right)\right)\\ \tau \left(x\right)=\frac{2\bar{\sigma }}{\phi \rho }K\mathrm{csch}\left(K\right)\cosh\left(K\left(1-\frac{2x}{l_{a}}\right)\right) \end{array}\right.$$

### 2.2. Unit Cell Elastic Modulus Analysis

Taking the unit cell as the basic building block of our biomimetic structure, then the structure stiffness can be defined by the material parameters and the geometrical parameters of the unit cells. Therefore, it is necessary to study the elastic modulus of the unit cell.

Following the definition of Hill [32], the volume-averaged stress $\bar{\sigma }=\frac{1}{V}\int_{V}\sigma dV$ and strain $\bar{\varepsilon }=\frac{1}{V}\int_{V}dV$ of a unit cell at both “phase 0” and “phase 1” were calculated separately, where V is the volume of a unit cell. Then, we obtained the effective elastic modulus ($\bar{E}$) of the unit cell, at “phase 0” and “phase 1”, denoted as ${\bar{E}}^{0}$ and ${\bar{E}}^{1}$, in terms of non-dimensional parameters.

At “phase 0”

(12) $${\bar{E}}^{0}=\phi E_{m}\frac{\left(\eta +2\right)\left[\alpha \eta +\left(\alpha +1\right)A\right]}{\left(\alpha +1\right)\eta +4A+\eta \left[\alpha \eta +\left(\alpha +1\right)A\right]}$$

where A=Ktanh(K) is a non-dimensional parameter for written simplicity.

At “phase 1”

(13) $${\bar{E}}^{1}=\phi E_{m}\frac{\left(\eta +2\right)A}{\left(\eta +4\right)A+\eta }$$

Combining ${\bar{E}}^{0}$ and ${\bar{E}}^{1}$

(14) $${\bar{E}}^{0}-{\bar{E}}^{1}=\frac{\phi E_{m}\alpha \left(\eta +2\right){\left(\eta +2A\right)}^{2}}{\left(\left(\eta +4\right)A+\eta \right)\left(\left(\alpha \eta +\eta +4\right)A+\left(\alpha \eta +\alpha +1\right)\eta \right)}>0$$

This formula indicates that the effective modulus of the unit cell must decline when the unit cell changes from “phase 0” to “phase 1”, and the decline ratio is written as below.

(15) $$\frac{{\bar{E}}^{1}}{{\bar{E}}^{0}}=\frac{\left(\eta +\frac{\left(\alpha +1\right)\eta +4A}{\alpha \eta +\left(\alpha +1\right)A}\right)A}{\eta +\left(\eta +4\right)A}$$

**Figure 4:**
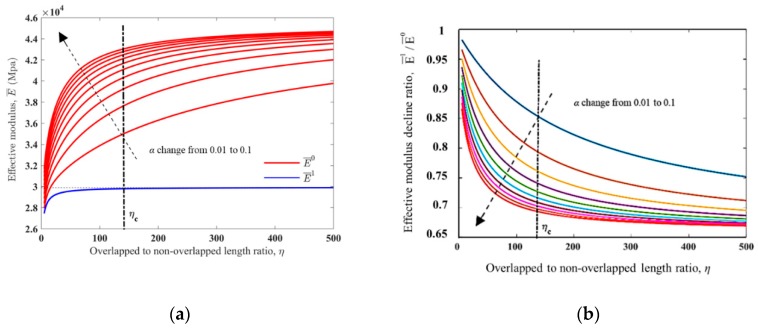
(**a**) The effective modulus of the unit cell at “phase 0” and “phase 1” and (**b**) the effective modulus decline ratio with respect to the non-dimensional geometrical parameter η and the non-dimensional material parameter α. In both (**a**) and (**b**), α changed from 0.01 to 0.1 discretely, as shown by the black dashed line. η were set from 5 to 500 continuously; the $\eta_{c}$ was marked with the black dash-dotted line.

Considering the convenience of the following simulation process and the improvement of the structure, the non-dimensional materials parameters α and β were referred to resin and rubber-like materials, which were widely used in the polyject method 3D-printing. On the other hand, the structural geometric configuration parameters are calculated by the theoretical model after the material parameters are determined, which can clearly reflect the changing trends of the equivalent elastic modulus and structural toughness of the designed structure. We chose the non-dimensional geometrical parameter η and the non-dimensional material parameter α as the independent variables, and studied their influence on ${\bar{E}}^{0}$, ${\bar{E}}^{1}$ and the effective modulus decline ${\bar{E}}^{1}/{\bar{E}}^{0}$. Besides α and η, other non-dimensional parameters used for plotting Figure 4 are listed in Table 1.

It is shown in Figure 4a that, as η increases, both ${\bar{E}}^{0}$ and ${\bar{E}}^{1}$ would increase with an attenuated increase rate, and finally approach their limiting value, derived by Equations (16) and (17) respectively. We particularly defined several critical values of $\eta_{c}$ upon differtent paramaters. First of all, we denoted the critical value of η for ${\bar{E}}^{1}$ as $\eta_{c}$. As η is the only influence factor on ${\bar{E}}^{1}$, we decided on $\eta_{c}$ as the baseline value. When $\eta =\eta_{c}$, ${\bar{E}}^{1}$ would reach 99.7% of its limiting value, which means η would have no more influence on ${\bar{E}}^{1}$ when $\eta \geq \eta_{c}$. With our definition, $\eta_{c}$ can be derived by Equation (18).

(16) $$\underset{\eta \to \infty }{\lim}{\bar{E}}^{0}=\phi E_{m}$$

(17) $$\underset{\eta \to \infty }{\lim}{\bar{E}}^{1}=\phi E_{m}\frac{A}{A+1}$$

(18) $$\begin{array}{ccc} \frac{\left(\eta_{c}+2\right)A}{\left(\eta_{c}+4\right)A+\eta_{c}} & = & 0.997\frac{A}{A+1}\\ \eta_{c} & = & \frac{662.667A-666.667}{A+1} \end{array}$$

However, as also shown in Figure 4a, ${\bar{E}}^{0}$ would be influenced by not only η but also α; ${\bar{E}}^{0}$ would increase as α increased from 0.01 to 0.1. Similarly, we denoted the critical value of η for ${\bar{E}}^{0}$ as $\eta_{c}\left({\bar{E}}^{0}\right)$, derived as Equation (19), which is influenced by α.

(19) $$\begin{array}{c} 0.997=\frac{\eta_{c}\left({\bar{E}}^{0}\right)+2}{\frac{\left(\alpha +1\right)\eta_{c}\left({\bar{E}}^{0}\right)+4A}{\alpha \eta_{c}\left({\bar{E}}^{0}\right)+\left(\alpha +1\right)A}+\eta_{c}\left({\bar{E}}^{0}\right)}\\ \eta_{c}\left({\bar{E}}^{0}\right)=\frac{0.167}{\alpha }\left(\begin{array}{c} 997-3A-1003\alpha -3A\pm \\ \sqrt{{\left(-997+3A+1003\alpha +3A\alpha \right)}^{2}-12\alpha \left(-1988A+2000A\alpha \right)} \end{array}\right) \end{array}$$

As shown in Figure 4b, the effective modulus decline ${\bar{E}}^{1}/{\bar{E}}^{0}$ would decrease as η increased, and finally approach the limiting value, derived by Equation (20). However, contrary to ${\bar{E}}^{0}$, ${\bar{E}}^{1}/{\bar{E}}^{0}$ will decrease as α increases from 0.01 to 0.1. Meanwhile, the critical value of η for ${\bar{E}}^{1}/{\bar{E}}^{0}$, i.e., $\eta_{c}\left({\bar{E}}^{1}/{\bar{E}}^{0}\right)$, is also influenced by α and derived as Equation (21).

(20) $$\underset{\eta \to \infty }{\lim}\frac{{\bar{E}}^{1}}{{\bar{E}}^{0}}=\frac{A}{A+1}$$

(21) $$\begin{array}{c} 0.997\frac{1}{A+1}=\frac{\eta_{c}\left({\bar{E}}^{1}/{\bar{E}}^{0}\right)+\frac{\left(\alpha +1\right)\eta_{c}\left({\bar{E}}^{1}/{\bar{E}}^{0}\right)+4A}{\alpha \eta_{c}\left({\bar{E}}^{1}/{\bar{E}}^{0}\right)+\left(\alpha +1\right)A}}{\eta_{c}\left({\bar{E}}^{1}/{\bar{E}}^{0}\right)+\left(\eta_{c}\left({\bar{E}}^{1}/{\bar{E}}^{0}\right)+4\right)A}\\ \eta_{c}\left({\bar{E}}^{1}/{\bar{E}}^{0}\right)=\frac{0.5015}{A\alpha }\left(\begin{array}{c} 1+A-0.997A^{2}+\alpha -2.988A\alpha -0.997A^{2}\alpha \pm \\ \sqrt{\left(\begin{array}{c} 3.988A\alpha (4A+0.012A^{2}-3.988A^{2}\alpha )+\\ {\left(1+A-0.997A^{2}+\alpha -2.988A\alpha -0.997A^{2}\alpha \right)}^{2} \end{array}\right)} \end{array}\right) \end{array}$$

Usually, in order to realize a large tunable stiffness range in our biomimetic structure, a small ${\bar{E}}^{1}/{\bar{E}}^{0}$ for the unit cell should be expected. Therefore with certain material components, (i.e., α is defined), the lower limit of η, i.e., $\eta_{c}\left({\bar{E}}^{1}/{\bar{E}}^{0}\right)$, will be determined by the demand of ${\bar{E}}^{1}/{\bar{E}}^{0}$.

### 2.3. Unit Cell Phase Changing Process Analysis

With the “joint part” breakage, the unit cell will change from “phase 0” to “phase 1” and the mechanical properties of the unit cell will change. Starting with the strength limit of the “joint part”, we further studied the relationship between stress and strain during the unit cell phase changing process.

We defined the limiting tensile stress of the “joint part” as $\sigma_{e}^{s}$, the limiting tensile stress of the “hard plate” as $\sigma_{m}^{s}$, and the limiting shear stress of “Region 3” as $\tau^{s}$. In order to fulfill the assumption that the “joint part” is the only breakable part under our design, the strength parameters within the unit cell ($\sigma_{e}^{s}$, $\sigma_{m}^{s}$ and $\tau^{s}$) should satisfy the following relationships

(22) $$\left\{ \begin{array}{l} \frac{\sigma_{5}}{\sigma_{e}^{s}}>\frac{\sigma_{4}}{\sigma_{m}^{s}}\\ \frac{\sigma_{5}}{\sigma_{e}^{s}}>\frac{\tau_{\max}}{\tau^{s}} \end{array}\right.$$

taking volume-averaged stress $\bar{\sigma }$ as the dependent variable and volume-averaged strain $\bar{\varepsilon }$ as the independent variable. Before the “joint part” breakage, the unit cell at “phase 0” should deform under the tensile load with the effective modulus ${\bar{E}}^{0}$. Then, we defined the critical average stress ${\bar{\sigma }}_{cr}^{0}$: when $\bar{\sigma }\leq {\bar{\sigma }}_{cr}^{0}$, the breakage will not happen and the unit cell at “phase 0”. ${\bar{\sigma }}_{cr}^{0}$ could be derived by combining Equation (7) with $\sigma_{e}^{s}$:

(23) $${\bar{\sigma }}_{cr}^{0}=\sigma_{e}^{s}\phi \frac{\alpha \eta +\left(\alpha +1\right)A}{\alpha \eta +2\alpha A}$$

Further, we derived the critical average strain $\bar{\varepsilon_{cr}}$, which represents the volume-averaged strain $\bar{\varepsilon }$ of the unit cell when the phase-change is just about to happen. With the “generalized Hooke law” at “phase 0”, $\bar{\varepsilon_{cr}}$ was derived as the following

(24) $$\bar{\varepsilon_{cr}}=\frac{{\bar{\sigma }}_{cr}^{0}}{{\bar{E}}^{0}}=\frac{\sigma_{e}^{s}}{E_{m}}\frac{\left(\alpha +1\right)\eta +\alpha \eta^{2}+\left(\alpha \eta +\eta +4\right)A}{\alpha \left(\eta^{2}+2\right)+2\alpha \left(\eta +2\right)A}$$

Considering that, when the “joint part” breaking, the shape of the unit cell must not abruptly change, i.e., saltation is impossible for the strain field, we concluded that $\bar{\varepsilon_{cr}}$ at “phase 0” is the same to that at “phase 1”. Therefore, for the equilibrium of the unit cell, the saltation must happen within the stress field, which means the critical average stress at “phase 1” (${\bar{\sigma }}_{cr}^{1}$) should not equal ${\bar{\sigma }}_{cr}^{0}$, i.e., ${\bar{\sigma }}_{cr}^{1}\neq {\bar{\sigma }}_{cr}^{0}$. Then, ${\bar{\sigma }}_{cr}^{1}$ can be derived along with the “generalized Hooke law” at “phase 1”, as in the following:

(25) $${\bar{\sigma }}_{cr}^{1}=\bar{\varepsilon_{cr}}{\bar{E}}^{1}=\sigma_{e}^{s}\phi \left[\frac{A}{1+A}+\frac{\left(1-\alpha \right)A}{2\alpha A+\alpha \eta }+\frac{2A^{2}-A}{\left(\eta +4\right)A^{2}+2\left(\eta +2\right)A+\eta }\right]$$

**Figure 5:**
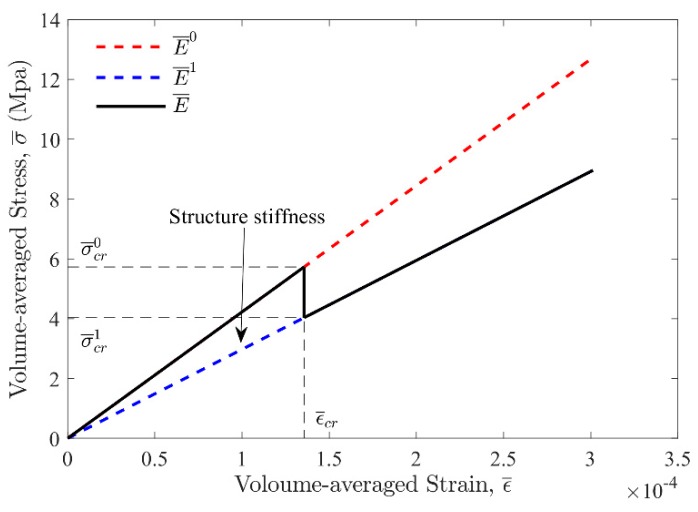
The volume-averaged stress–strain curve for the unit cell during “phase changing”, which is marked with the black line. The effective elastic moduli at “phase 0” and “phase 1” are marked with the red dashed line and blue dashed line, respectively. The critical average strain, $\bar{\varepsilon_{cr}}$, and the critical average stress, ${\bar{\sigma }}_{cr}^{0}$, as well as ${\bar{\sigma }}_{cr}^{1}$, is marked with black dashed line. The structure stiffness of the unit cell defined in this paper is encircled by the black line and the blue dashed line.

After changing into “phase 1”, the unit cell will deform with the effective elastic modulus ${\bar{E}}^{1}$, until reaching the ultimate strength limit of the unit cell (decided by $\sigma_{m}^{s}$ and *τ^s^*). With all the characteristic parameters derived above, we obtained the volume-averaged stress–strain relationship of the whole “phase changing” process, as shown in Figure 5.

### 2.4. Structure Toughness Analysis

While the term “toughness” has multiple usages, the “structure toughness” mentioned in this paper is not the material toughness (usually defined as the maximum energy adsorbed per mass before fracture [19]). Instead, we explored hierarchical and structure parameter-dependent toughness—e.g., “structure toughness”. This particular toughness is defined as the specific energy dissipation needed for the breakage of the “joint parts”, which also reflected the extra toughness increase for our biomimetic structure during “phase changing”, compared with the classical plate-staggered structure.

We neither made assumptions of “joint part” reformation nor a reversible process. Therefore, the stress-strain response is unidirectional, and no hysteretic behavior needs considering. We denoted the normalized “structure toughness” of a unit cell as $T_{cell}$, and defined it as the following:

(26) $$T_{cell}=\frac{\int_{V}\bar{\varepsilon_{cr}}\left({\bar{\sigma }}_{cr}^{0}-{\bar{\sigma }}_{cr}^{1}\right)dV}{2\int_{V}dV}$$

Noting that the “joint part” breaking event results in a repeated pattern within every unit cell, the “structure toughness” T for the whole biomimetic structure can be simply summarized as:

(27) $$T=\sum T_{cell}$$

Therefore, without loss of generality, we took the unit cell as before, and analyzed its $T_{cell}$. Substituting Equations (23)–(25) into Equation (26), then $T_{cell}$ can be derived as:

(28) $$T_{cell}=\phi \frac{\sigma_{e}^{s}{}^{2}}{E_{m}}\frac{\eta \left(1+\eta +\frac{1}{\alpha }\right)+A\left(\frac{4+\eta }{\alpha }+\eta \right)}{2\left(2+\eta \right)\left(\eta +A\left(4+\eta \right)\right)}$$

**Figure 6:**
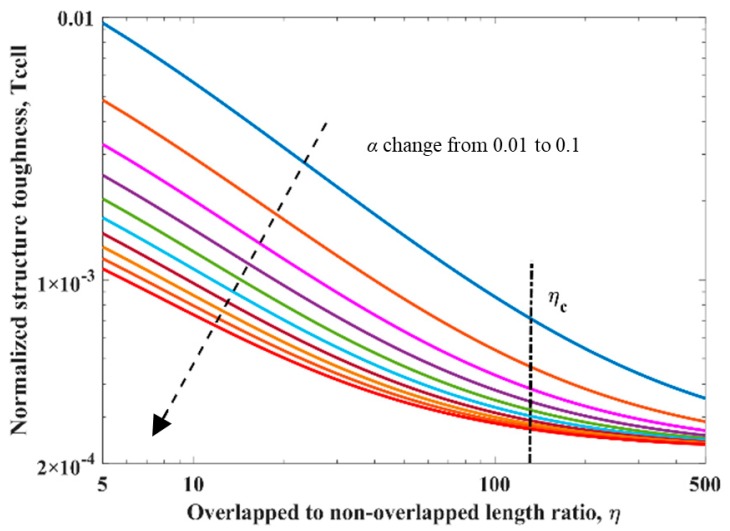
The structure toughness change with respct to the non-dimensional geometrical parameter *η* and the non-dimensional material parameter α, in the logarithmic coordinate. α changed from 0.01 to 0.1 discretely, shown with a black dashed line. η changed from 5 to 500 continuously, and the $\eta_{c}$ is marked with a black dash-dotted line. Other parameters used for making this figure were inherited from Table 1.

As is shown in Figure 6 with η increasing, the normalized structure toughness of the unit cell $T_{cell}$ would decrease with an attenuated decrease rate, and finally approach the limiting value, written as Equation (29). As α increases from 0.01 to 0.1, $T_{cell}$ would decrease, and the critical value of η for $T_{cell}$ i.e., $\eta_{c}\left(T_{cell}\right)$, derived by Equation (30), is also influenced by α.

(29) $$\underset{\eta \to \infty }{\lim}T_{cell}=\frac{\phi \sigma_{e}^{s}{}^{2}}{2E_{m}}$$

(30) $$\begin{array}{c} 0.997=\frac{\eta_{c}\left(T_{\mathrm{cell}}\right)\left(1+\eta_{c}\left(T_{\mathrm{cell}}\right)+\frac{1}{\alpha }\right)+A(\frac{4+\eta_{c}\left(T_{\mathrm{cell}}\right)}{\alpha }+\eta_{c}\left(T_{\mathrm{cell}}\right))}{\left(2+\eta_{c}\left(T_{\mathrm{cell}}\right))(\eta_{c}\left(T_{\mathrm{cell}}\right)+A(4+\eta_{c}\left(T_{\mathrm{cell}}\right))\right)}\\ \eta_{c}\left(T_{cell}\right)=\frac{\left(\begin{array}{c} l+A-0.994\alpha -4.982A\alpha \pm \\ \sqrt{{\left(\frac{A+l}{\alpha }-0.994-4.982A\right)}^{2}+\left(3.988A-0.012)(\frac{4A}{\alpha }-7.976A\right)} \end{array}\right)}{1.994A\alpha -0.006\alpha } \end{array}$$

Moreover, to gain a high $T_{cell}$ for the unit cell, both α and η should be set as small as possible, under the compromise of ${\bar{E}}^{1}/{\bar{E}}^{0}$ and the design constraints. Therefore, with certain material components (i.e., α is defined), the upper limit of η, i.e., $\eta_{c}\left(T_{cell}\right)$, for the unit cell will be determined by *T_cell_* requirement.

## 3. Finite Element Modeling and Simulation

We validated the generality and accuracy of our mechanical model by finite element analysis (FEA) with ABAQUS/CAE, steady-state static, direct solver. Then, we compared the simulation results and the theoretical results of the stiffness decline ${\bar{E}}^{1}/{\bar{E}}^{0}$, as well as the structure toughness *T_cell_* of the unit cell.

### 3.1. FEM Simulation Preparation

**Figure 7:**
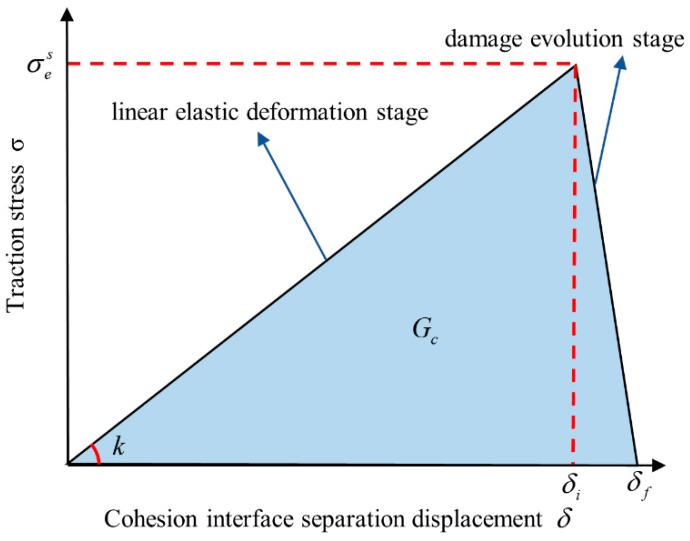
Bilinear cohesive damage model.

The cohesion FEM model can simulate the failure process of materials by introducing the cohesion model within the materials. In this paper, zero-thickness cohesion elements COH2D4 were inserted between every solid elements CPE4R element at Region 5 to simulate the designed progressive damage and breaking process of the “joint part” under external load. The zero thickness COH2D4 element can be regard as two connecting faces of adjacent solid elements. The cohesive elements describe the damage and failure, which have different forms, by the “traction separation law”. To remain consistent with the theoretical analysis, we chose the “bilinear cohesive damage model” in the ABAQUS platform, as shown in Figure 7. Under external load, the cohesive element would firstly go through the elastic deformation stage, and when meeting the damage initiation criteria, the damage evolution stage would begin, and, after the cohesive element completely failed, it would be deleted. 

In Figure 7, the abscissa and ordinate are, respectively, the cohesive element separation displacement and the traction stress; the slope of the elastic deformation stage is the cohesive element stiffness k; $\delta_{i}$ is the separation displacement at damage initiation; and $\sigma_{e}^{s}$ is the traction stress at damage initiation, which is equivalent to the “maximum tensile stress” when the “Maxs” principle is selected as the cohesive element failure criterion in ABAQUS; $\delta_{f}$ is the failure displacement; and the triangle area enclosed by the elastic deformation stage curve, the damage evolution stage curve and the abscissa is the fracture energy $G_{c}$.

**Figure 8:**
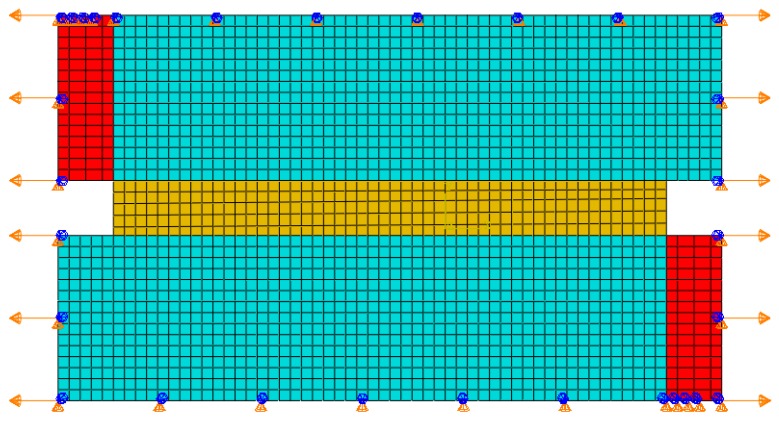
Finite element modelling (FEM) model with mesh properties of the unit cell. Cohesive elements are inserted in the red region. In the FEM model, the periodic boundary conditions were set to the upper and lower edges, and uniform-speed displacement tension load was set to the left and right edges.

The FEM model with mesh properties of the unit cell is shown in Figure 8. In order to keep consistent with theoretical analysis, isotropic linear elastic materials were chosen for all parts in the unit cell FEM model, and the “Maxs” principle was selected as the COH2D4 failure criterion. Moreover, the elastic modulus of the COH2D4 elements should be the same as the adjacent CPE4R elements. The geometrical and material properties used for the simulation are listed in Table 2 and Table 3, respectively. Considering the periodic property of the structure, the periodic boundary conditions were set to this unit cell FEM model, and the uniform-speed displacement load was applied to the left and right edges, as shown in Figure 8. 

### 3.2. Simulation Results and Discussion

**Figure 9:**
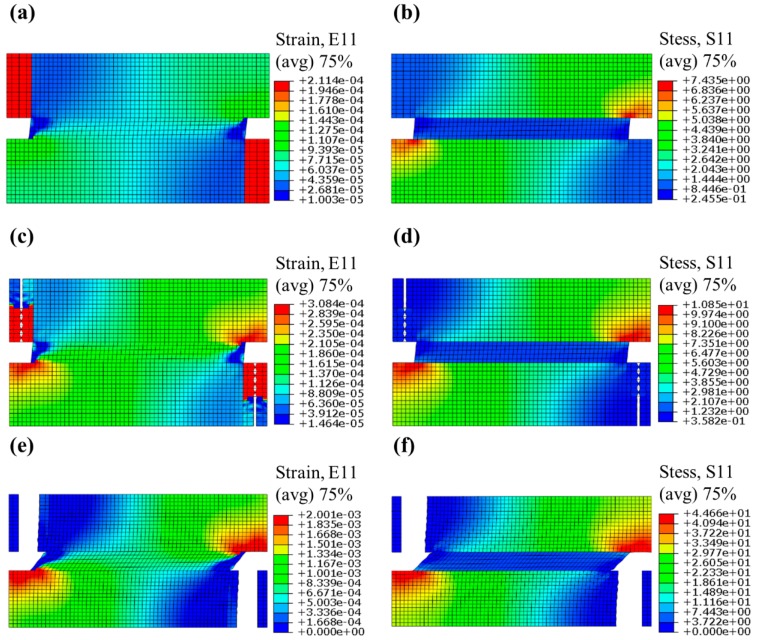
(**a**,**c**,**e**) strain and (**b**,**d**,**f**) stress contour figures of the deformed FEM model under displacement load. (**a**,**b**) are at phase “0”, (**e**,**f**) are at phase “1” and “b” “c” are during the Region 5 breaking process.

Under the displacement load, the Region 5 break, as designed, and the unit cell FEM model, went through the phase changing process. Figure 9a–f displays the strain and stress contour figures of the deformed FEM model, changing from “phase 0” to “phase 1” during simulation. Especially, the gradient change of stress and strain along y direction owe to the stress concentration that occurred at the intersection of Region 3 and Region 1 (2), which is relatively small compared with the gradient change along x direction, and is neglected while theoretical modeling and analyzing.

Figure 9b shows the stress distribution within the unit cell at “phase 0”, under uniaxial tensile, which basically coincides with the trend theoretically derived by Equation (5). Figure 9f shows the stress distribution of the unit cell at “phase 1”, which basically coincides with Equation (11).

**Figure 10:**
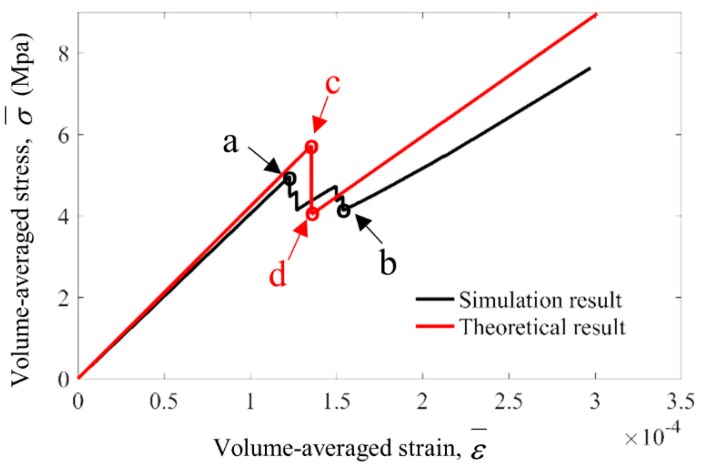
Comparison between FEM simulation and theoretical derivation of the volume-averaged stress–strain curve during the phase changing process. The volume-averaged strain and stress at feature points a, b, c, d are ${\bar{\varepsilon }}_{cr}^{a}=1.225\times 10^{-4}$, ${\bar{\sigma }}_{cr}^{a}=4.953$, ${\bar{\varepsilon }}_{cr}^{b}=1.541\times 10^{-4}$, ${\bar{\sigma }}_{cr}^{b}=4.137$$\bar{\varepsilon_{cr}^{c}}=\bar{\varepsilon_{cr}^{d}}=1.356\times 10^{-4}$, ${\bar{\sigma }}_{cr}^{c}=5.729$, ${\bar{\sigma }}_{cr}^{d}=4.033$.

As shown in Figure 10, the stress–strain relationship during FEM simulation agrees well with the theoretical result. The fluctuation of the simulation curve from point a to point b happened during the phase changing process. This is because the breakage of Region 5 would start at point a (${\bar{\varepsilon }}_{cr}^{a}=1.031\times 10^{-4}$) and gradually develop along y direction, as shown in Figure 9e,f, until the complete breakage at point b (${\bar{\varepsilon }}_{cr}^{b}=1.296\times 10^{-4}$), while in theoretical analysis we assumed the breakage would complete instantaneously at point c and point ($\bar{\varepsilon_{cr}^{c}}=\bar{\varepsilon_{cr}^{d}}=1.356\times 10^{-4}$). With this assumption, the relative errors of simulation results ${\bar{E}}^{0}$, ${\bar{E}}^{1}$, ${\bar{E}}^{1}/{\bar{E}}^{0}$ and $T_{cell}$ compared to the theoretically derived ${\bar{E}}^{0}$, ${\bar{E}}^{1}$, ${\bar{E}}^{1}/{\bar{E}}^{0}$ and $T_{cell}$ are shown in Table 4.

**Figure 11:**
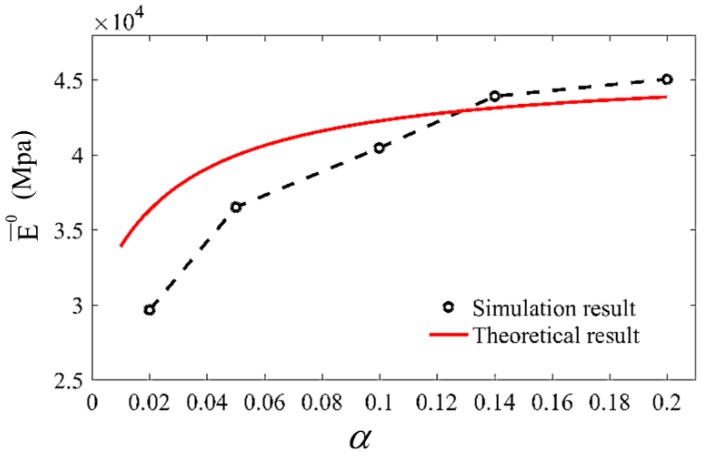
Comparison between FEM simulation and theoretical results of the effective modulus changing with α, while η=100. In FEM simulation, represented by black circles, α was set as 0.02, 0.05, 0.1, 0.14, 0.2, and the corresponding ${\bar{E}}^{0}$ were 2.9662 × 10^4^, 3.6505 × 10^4^, 4.046 × 10^4^, 4.3904 × 10^4^, 4.5037 × 10^4^.

Next, we simulated the influence of α on the unit cell effective modulus. In this simulation, α was set as 0.02, 0.05, 0.1, 0.14 and 0.2, by adjusting the material property $E_{e}$ successively, while η was kept as 100. The simulation result was compared with theoretical results, in which α continuously changed from 0.01 to 0.2, as shown in Figure 11. It is predicted by the theoretical analysis and verified by this simulation that, when η is set, as α increases, ${\bar{E}}^{0}$ would increase with an attenuated increase rate and finally approach a limiting value. As can be seen in the above analysis, the limited value should be defined by η.

## 4. Conclusions

In summary, we have brought up a new biomimetic composite structure with tunable stiffness and superior structure toughness via a designed progressive breakable constituent. We mainly focused on the periodic unit cell of the structure, established the mechanical model of the unit cell and verified it with FEM simulation. Two theoretical relations describing the elastic modulus decline ratio and the unit cell toughness promotion are developed as functions of the geometrical parameters and the material parameters, respectively. Moreover, we demonstrate a strategy to adjust the unit cell stiffness and structure toughness by typical geometrical parameter η (the “hard plate” overlapped length to non-overlapped length ratio) and typical material parameter α (the “joint part” tensile modulus to “hard plate” tensile modulus ratio).

Based on the above theatrical analysis and FEM simulation, we can draw the following conclusions. Firstly, the breakage of the “joint part” within the unit cell, while phase changing, does not mean biomimetic composite structure failure. The strength property of the structure should be decided at “phase 1”, by the tensile strength of “hard plate” and the shear strength of “shear part” together. Therefore, with the proper choice of materials for the “hard plate” and “shear part”, when the breakage of Region 5 occurs, the unit cell can realize extra energy dissipation and stiffness changing without loss of strength. Secondly, as η increases, both ${\bar{E}}^{0}$ (the effective modulus at “phase 0”) and ${\bar{E}}^{1}$ (the effective modulus at “phase 1”) would increase with an attenuated increase rate and finally approach a limiting value, derived as Equations (16) and (17). However, as α increasing, only ${\bar{E}}^{0}$ will be influenced, i.e., increase with an attenuated increase rate and finally approach a limiting value. Therefore, the structure stiffness before phase changing will restrict the material selection. Thirdly, ${\bar{E}}^{1}/{\bar{E}}^{0}$ (the effective modulus decline ratio) and $T_{cell}$ (the normalized “structure toughness” of a unit cell) are two unique target parameters for our design. They will respectively decide the lower and upper limiting value of η, derived as Equations (21) and (30), which are two constraint conditions for structure configuration design.

To recapitulate, the stiffness changing and toughness promotion of our biomimetic structure can be precisely achieved with theoretically calculated structure parameters, i.e., by quantitatively tailoring the “joint part” breaking process within each unit cell. The theoretical analysis was verified by the FEM simulation. Moreover, the simulation results provide more detailed insights into the mechanical properties of the unit cell, such as the detailed stress and strain field output and the specific breaking process of Region 5 during phase changing. Therefore, combining the theoretical predication and the FEM verification, it is possible to adjust the properties of our biomimetic composite structure. With superior toughness and tunable stiffness, our biomimetic composite structure can serve as a guideline in designing novel load-bearing structures.

Future extension of this study can involve investigating and designing the dynamic properties of the unit cell, under dynamic load. Taking the unit cell as the basic building block, a hierarchical structure can be constructed with different unit cell arrangement modes. Then, the influence of the unit cell properties, as well as the arrangement modes on the whole structure dynamic response, can be studied.

## Figures and Tables

**Table 1:** The value of the non-dimensional parameters set for analysis.

Non-Dimensional Parameters	η	α	β	ρ	λ	ϕ	*A*
Value	5–500	0.01–0.1	0.4	2	10	0.9091	1.928

**Table 2:** Geometrical properties used for FEM simulation.

Geometric Parameter	b	h	$l_{a}$	$l_{b}$
(mm)	3	1	10	1

**Table 3:** Materials properties used for FEM simulation.

Part	Element Type	E (MPA)	Poisson’s Ratio	$\sigma_{e}^{s}\;(\mathrm{Mpa})$	$G_{c}\;(N/\mathrm{mm})$
Hard plate	CPE4R	50000	0.3		
Shear part	CPE4R	5600	0.4		
Joint part	CPE4R	5000	0.3		
Joint part	COH2D4	5000	0.3	5	2 × 10^−2^

**Table 4:** Relative errors of FEM simulation and theoretical results.

	${\bar{E}}^{0}\;(\mathrm{Mpa})$	${\bar{E}}^{1}\;(\mathrm{Mpa})$	${\bar{E}}^{1}/{\bar{E}}^{0}$	$T_{cell}\;(\mathrm{mJ}/{\mathrm{mm}}^{3})$
Theoretical result	4.226 × 10^4^	2.975 × 10^4^	0.704	1.420 × 10^−4^
Simulation result	4.046 × 10^4^	2.565 × 10^4^	0.6341	1.446 × 10^−4^
Relative error	4.27%	13.77%	9.92%	1.84%
